# Successes and challenges towards improving quality of primary health care services: a scoping review

**DOI:** 10.1186/s12913-023-09917-3

**Published:** 2023-08-23

**Authors:** Aklilu Endalamaw, Resham B Khatri, Daniel Erku, Frehiwot Nigatu, Anteneh Zewdie, Eskinder Wolka, Yibeltal Assefa

**Affiliations:** 1 School of Public Health, the University of Queensland, Brisbane, Australia; 2 College of Medicine and Health Sciences, Bahir Dar University, Bahir Dar, Ethiopia; 3 Health Social Science and Development Research Institute, Kathmandu, Nepal; 4 Centre for Applied Health Economics, School of Medicine, Griffith University, Griffith, Australia; 5 Menzies Health Institute Queensland, Griffith University, Griffith, Australia; 6 International Institute for Primary Health Care in Ethiopia, Addis Ababa, Ethiopia

**Keywords:** Quality, Primary health care, Donabedian model

## Abstract

**Background:**

Quality health services build communities’ and patients’ trust in health care. It enhances the acceptability of services and increases health service coverage. Quality primary health care is imperative for universal health coverage through expanding health institutions and increasing skilled health professionals to deliver services near to people. Evidence on the quality of health system inputs, interactions between health personnel and clients, and outcomes of health care interventions is necessary. This review summarised indicators, successes, and challenges of the quality of primary health care services.

**Methods:**

We used the preferred reporting items for systematic reviews and meta-analysis extensions for scoping reviews to guide the article selection process. A systematic search of literature from PubMed, Web of Science, Excerpta Medica dataBASE (EMBASE), Scopus, and Google Scholar was conducted on August 23, 2022, but the preliminary search was begun on July 5, 2022. The Donabedian’s quality of care framework, consisting of structure, process and outcomes, was used to operationalise and synthesise the findings on the quality of primary health care.

**Results:**

Human resources for health, law and policy, infrastructure and facilities, and resources were the common structure indicators. Diagnosis (health assessment and/or laboratory tests) and management (health information, education, and treatment) procedures were the process indicators. Clinical outcomes (cure, mortality, treatment completion), behaviour change, and satisfaction were the common indicators of outcome. Lower cause-specific mortality and a lower rate of hospitalisation in high-income countries were successes, while high mortality due to tuberculosis and the geographical disparity in quality care were challenges in developing countries. There also exist challenges in developed countries (e.g., poor quality mental health care due to a high admission rate). Shortage of health workers was a challenge both in developed and developing countries.

**Conclusions:**

Quality of care indicators varied according to the health care problems, which resulted in a disparity in the successes and challenges across countries around the world. Initiatives to improve the quality of primary health care services should ensure the availability of adequate health care providers, equipped health care facilities, appropriate financing mechanisms, enhance compliance with health policy and laws, as well as community and client participation. Additionally, each country should be proactive in monitoring and evaluation of performance indicators in each dimension (structure, process, and outcome) of quality of primary health care services.

**Supplementary Information:**

The online version contains supplementary material available at 10.1186/s12913-023-09917-3.

## Introduction

Quality of care is the extent to which the health care system can achieve the desired health care goals, such as effective recovery, preventing premature mortality, halting disease progression from being complicated, and maximising clients’ satisfaction with the care they received [1]. With efficient, integrated, equitable, timely, people-centred, and safe health services, preventive and promotive, treatment, palliative, and rehabilitative quality care could be achieved [2]. These services are provided in primary health care (PHC) [3], for which quality is an attribute in the first-contact care of several health conditions [4]. Because PHC is planned to deliver essential health services as close to home as possible, it serves as a roadmap to universal health care coverage (UHC), which must be of high quality to achieve the health system’s vision.

Quality is currently on the agenda of sustainable development goals that target UHC [5]. The World Health Organisation (WHO), the Organisation for Economic Co-operation and Development, and the World Bank emphasised that ensured quality is a fundamental component of UHC [6]. To streamline policy and PHC quality implementation, a series of national strategic directions have been adopted [7]. Notable quality and safety standards or strategies have been established in some countries, for example, Australia [8, 9], European [10] and African countries [11]. Good health governance and administration [12], quality improvement programmes [13], financial and non-financial support, community empowerment and engagement, competent health care providers, and monitoring and evaluation [14] are some of the quality improvement strategies. These schemes have a vital role in improving the patient experience in PHC, including quality of care, satisfaction, and the health of populations [15].

Despite these strategies, poor-quality care is a continuing public health debate. This could be explained by safety problems, a large percentage of hospital-acquired infections, a high burden of amenable mortality, and excess health care expenditure. Globally, the estimated annual cost due to medication errors is 42 billion United States dollars (US$) [16]. Similarly, more than 10% of hospital expenditure in high-income countries is due to medical errors or hospital-acquired infections [17], where 1 in 10 patients experience medical errors while receiving hospital care, and 7 out of 100 hospitalised patients (1 in 10 in developing countries) acquire a health care-associated infections [17, 18]. This situation is recorded much more unacceptable, especially in less developed countries. A systematic analysis of preventable deaths in 137 low- and middle-income countries (LMICs) revealed that 5.0 million deaths are attributed to poor-quality care annually [19], which imposes costs of US$ 1.4 to 1.6 trillion each year in lost productivity [20].

The health system could prevent many deaths if high-quality care were implemented. The Lancet Global Health Commission estimated that high-quality health systems could prevent 8 million deaths yearly in LMICs [5]. This requires systematic and coherent evidence-based actions that give emphasis quality [21] that pragmatic framework can measure.

Donabedian’s quality of care measurement model is considered a logical quality measurement framework to produce evidence on quality care based on the structure, process, and outcome dimensions [22]. This framework indicates what systems, policies, and infrastructure should be in place to ensure the delivery of high-quality PHC services towards the most desired health care outcome. This helps to identify challenges that need improvement, including commenting on the presence of policy documents or workable guidelines and the interaction between clients and health care providers. Experts advise that it is crucial to measure quality of care with a focus on the interaction between structure, process, and outcome dimensions because outcome status reflects the structure and process indicators [23]. The WHO’s ‘Network for Improving Quality of Care Programme’ has identified four measures for improving quality of health care. These are patient outcome measures, patient process measures, facility input or structure-related measures, and programme performance measures [24]. Identifying crucial quality indicators in health care provision is also suggested [25].

Previous reviews focused on either individual countries or specific diseases only. For example, a review on depression [26] and outpatient practise of primary care in the United States of America (USA) and the United Kingdom (UK) [27] did not address the successes and challenges in providing quality care in the PHC system. Another review focused on the quality indicators of PHC and also did not address the successes and challenges of quality of care [28]. Therefore, scoping all available evidence, including original articles, reviews, professional discussions, or arguments, will provide information for researchers and highlight areas for policy and decision makers to take corrective action on the identified gaps. This scoping review summarised indicators, successes, and challenges in delivering quality PHC services.

## Methods

### Search strategy

This review is guided by the preferred reporting items for systematic reviews and the meta-analysis extension for scoping reviews (PRISMA-ScR) to adhere to procedural activities starting from search strategy to reporting findings [29]. A systematic search of literature from databases was conducted between 05 July 2022 and 23 August 2022 with no date restriction to access articles from inception to the final search date. Then, the screening process proceeded after fully-exported all articles into EndNote x9 reference manager software. The databases we accessed to identify articles were PubMed, Web of Science, Excerpta Medica dataBASE (EMBASE), and Scopus. We also searched Google Scholar to find additional literature. We operationalised the concept of quality of care in this study using Donabedian’s model [22]. The Donabedian model addresses structure (availability of inputs and resources, appropriateness of facilities and administration), process (indicators streamlined from patient and health worker interaction), and outcome (interventions’ health effects). Search terms were “primary health care”, “primary healthcare”, “primary care”, “quality of care”, quality, “quality care”, “quality of health care”, “quality of healthcare”, Donabedian, “Donabedian’s model”, “Donabedian model”, “Donabedian’s structure process outcome”, “Donabedian’s structure-process-outcome”, “Donabedian structure process outcome” and “structure process outcome”. Different Boolean operators were used. These are: “AND” and “OR” to expand or narrow the search parameters, quotation marks (“”) to get results with the exact phrases; and parentheses to group search terms. The search strategy fitted in PubMed was (((((“primary health care” [All Fields] OR “primary healthcare”[All Fields] OR “primary care”[All Fields]) AND “quality of care”[All Fields]) OR “quality”[All Fields] OR “quality care”[All Fields] OR “quality of health care”[All Fields] OR “quality of healthcare”[All Fields]) AND “Donabedian”[All Fields]) OR “Donabedian’s model”[All Fields] OR “Donabedian’s structure process outcome”[All Fields] OR “Donabedian model”[All Fields]) OR “Donabedian structure process outcome”[All Fields] OR “Donabedian’s structure-process-outcome”[All Fields] OR “Donabedian structure-process-outcome”[All Fields] OR “structure-process-outcome”[All Fields]. The search strategy for Scopus, Web of Science and EMBASE is available in the supplementary file 1.

### Selection criteria and data extraction

Searches were limited to articles published in English. We used ‘population’, ‘concept’ and ‘context’ frameworks to establish a search strategy and include articles [30]. The population was any participants, PHC personnel (general practitioners, nurses, pharmacies, midwives, dentists, etc.), or clients who participated in the study. The ‘concept’ was the quality of PHC, which approached Donabedian’s structure-process-outcome model. The ‘context’ was any study setting, including urban or rural institutions (district hospitals, health centres), community care, nursing homes, family care, or if articles mentioned PHC settings in any country. When articles did not mention PHC, we reviewed keywords, and included the article if it fulfilled other criteria. The search was tailored to any document type, such as an article, review, perspective, opinion, letter, commentator, etc. However, we only found opinions, professional discussion, reviews, and articles. Previous reviews have reported the synthesis from different original studies, which may not be necessarily conducted by the Donabedian input-process-output framework, but the reviews should summarise the findings into this framework context to be included in the current review. The reference lists of previous reviews were assessed to check whether original studies included in the review were conducted based on Donabedian framework. Primary studies included in the review articles were in different contexts, dimensions, types of cases, functions, and domains except one review for from 2005 [31], which is included in another from 2010 [32]. Therefore, we could not directly include the primary studies that were included in the former reviews except these two reviews 2005 and 2010 [31, 32]. We decided to include both reviews because only part of information from the 2005’s review [31] included in the 2010 [32]. Additionally, one of the purposes of a scoping review is to include any type of article, including previous reviews, to map the available literature besides summarising results [33]. Therefore, the steps before data extraction were article search, exporting all accessed articles into EndNote x9 reference manager, duplication check, screening articles for title, screening articles for abstract, and full-text assessment. Author, publication year, country discussed, type of study or study design, PHC setting, study participant, and main findings of included documents were extracted.

### Data synthesis

The main findings for structure, process, and outcome dimensions were synthesised using a narrative approach. Success was defined as high-quality care or improved quality of care. Any observed gap in the quality of PHC or barriers that affected the provision of quality of PHC were narrated as challenges. The search and characteristics of results, PHC quality indicators, successes, and challenges of quality in PHC were described sequentially in the result section. Summary of professional discussion: neither success nor challenges were described in the PHC quality indicators section of the result.

## Results

### Search results

**Fig. 1:**
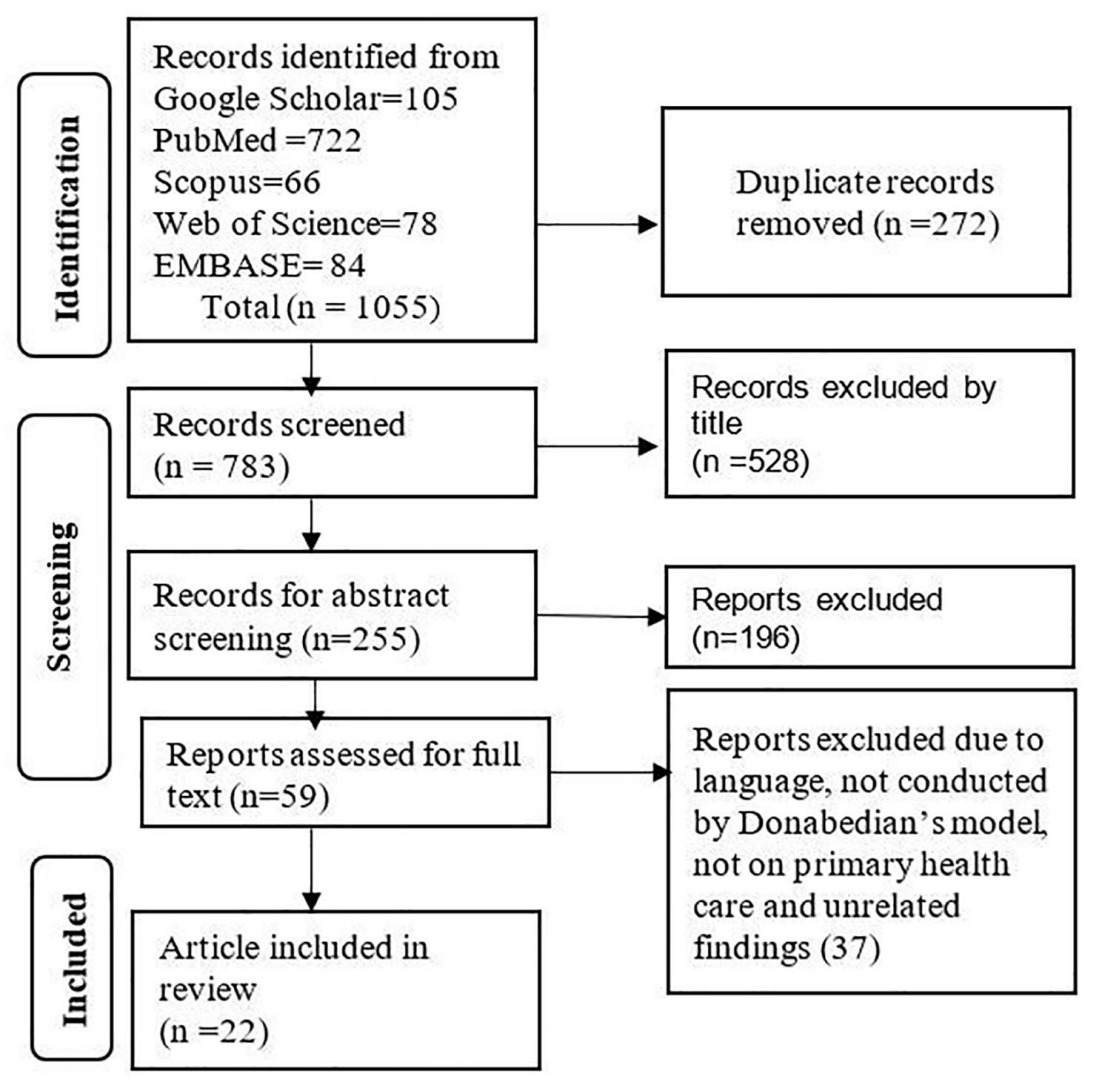
PRISMA article selection process adapted from PRISMA 2020 for new systematic reviews which included searches of databases

A total of 1,055 documents were available. These articles were accessed using the final search strategy of Web of Science (84 articles), Scopus (66 articles), and PubMed (722 articles), as well as searching of articles by topic in Google Scholar (105). The final articles (1,055) were exported in EndNote X9 and checked for duplication. After we removed duplication (272 were excluded), 783 were eligible for title screening. A total of 528 were excluded by title screening. Then, 255 were eligible for abstract screening, and 196 were excluded due to the abstract not having information related to the objectives. Then, 59 articles were eligible for full text screening, and 37 were excluded. Finally, 22 were eligible for the current result synthesis (Fig. 1).

### Characteristics of articles

Three articles were from Japan [34–36], two each from the USA [32, 37], South Africa [38, 39], Ethiopia [40, 41], and Iran [42, 43]. Brazil [44], Canada [31], Nigeria [45], Uganda [46], LMICs [47], and upper-middle- and high-income countries [28] each had one. Others were from two or more high-income countries [26, 27, 48]. One author published an opinion article on the structure, process, and outcome dimensions of primary dental care, which was not specific to any country [25]. Another author discussed the definition and framework based on the context of the UK, New Zealand, and Germany [23]. Overall, ten articles were from high-income countries [23, 26, 27, 31, 32, 34–37, 48], three articles were from upper-middle-income countries [38, 39, 44], four articles were from LMICs [42, 43, 45, 47], three were from low-income countries [40, 41, 46], and one each upper-middle- and high-income countries [28] and not-specific [25].

Regarding article type, seven were different types of reviews [26–28, 31, 32, 47, 48], and five were cross-sectional studies [38, 41–43, 46]. Others were qualitative studies [34, 36, 39], mixed-method studies [40, 45], Delphi study [35], evaluation study [44], retrospective cohort study [37], opinion [25], and an operational discussion [23].

The included articles focused on several health problems. Eight articles focused on the overall PHC settings [26–28, 35, 41, 44, 47, 48]. Four articles were on nursing home care [31, 32, 36, 42], three studies were conducted each in rural health care settings [37–39], and health care centres [43, 45, 46], and one each in district public health facilities [40], and community pharmacy [34].

Four articles were focused on chronic diseases [38, 39], including mental health problems [26, 32] and diabetes [42]. One article on both chronic and communicable diseases [37]. Three articles focused on women’s health: early abortion care [47], antenatal care/ANC/ [41], and preconception care [43]. The other three articles were on pharmaceutical services [34–36]. The remainders were on tuberculosis [46], oral health care [44], youth-friendly health services [40], osteoarthritis [48], dental care [25], and not specific diseases [23, 27, 28, 31, 45].

### PHC quality indicators

Several indicators were identified in the structure, process, and outcome dimensions of PHC quality.

Byrne and Tickle argue in their opinion article that six domains of health care quality—safety, effectiveness, timeliness, patient-centredness, efficiency, and equitability—have to be measured for structure, process, and outcome to assess the quality of primary dental care [25]. Gardner and Mazza, who explored implementing of the quality framework in general practise settings in New Zealand, the UK, Germany, and Australia, concluded that the application of the Donabedian framework varies across countries [23]. An umbrella review identified 727 PHC quality indicators: 74.5% were process indicators, 19.2% were outcome indicators, and the remainder (6.3%) were structure indicators, and these indicators were related to safety, effectiveness, timeliness, patient-centredness, efficiency, and equitability [28].

Other reviews identified quality indicators, which were 134 on geriatric pharmacotherapy [35], 53 on depression [26], 21 on early abortion care [47], and 20 on osteoarthritis [48]. The types or numbers of indicators depend on the nature of the disease. For example, 80% and 38% of indicators were related to treatment safety and causes of drug selection in geriatric pharmacotherapy, respectively [35], and the majority (82%) of quality indicators were process indicators in this therapy [35]. There was no structured indicator for the quality measurement of geriatric pharmacotherapy delivered by community pharmacists [35]. From 53 quality indicators, 16 structure, 33 process, and 4 outcome indicators were identified in depression care; a “do not do” process indicator for some selected antidepressant drugs was identified [26]. As an additional example, the 20 quality indicators (2 structure, 16 process, and 2 outcome domains) in osteoarthritis care are further grouped into two structures, nine processes, and two outcome indicators [48]. According to the home health care professional’s perspective, home pharmaceutical care were established with 9 themes and 27 subthemes [36]. One study discussed the Donabedian care model as a mediation pathway; structure indicators can directly affect outcome indicators [38].

In few studies, some process determinants were grouped into structural indicators. To illustrate, waiting time [48], teamwork [34, 36], and professionalism [34, 36] were reported in the structure domain, but they are also involved in the process domain.

**Fig. 2:**
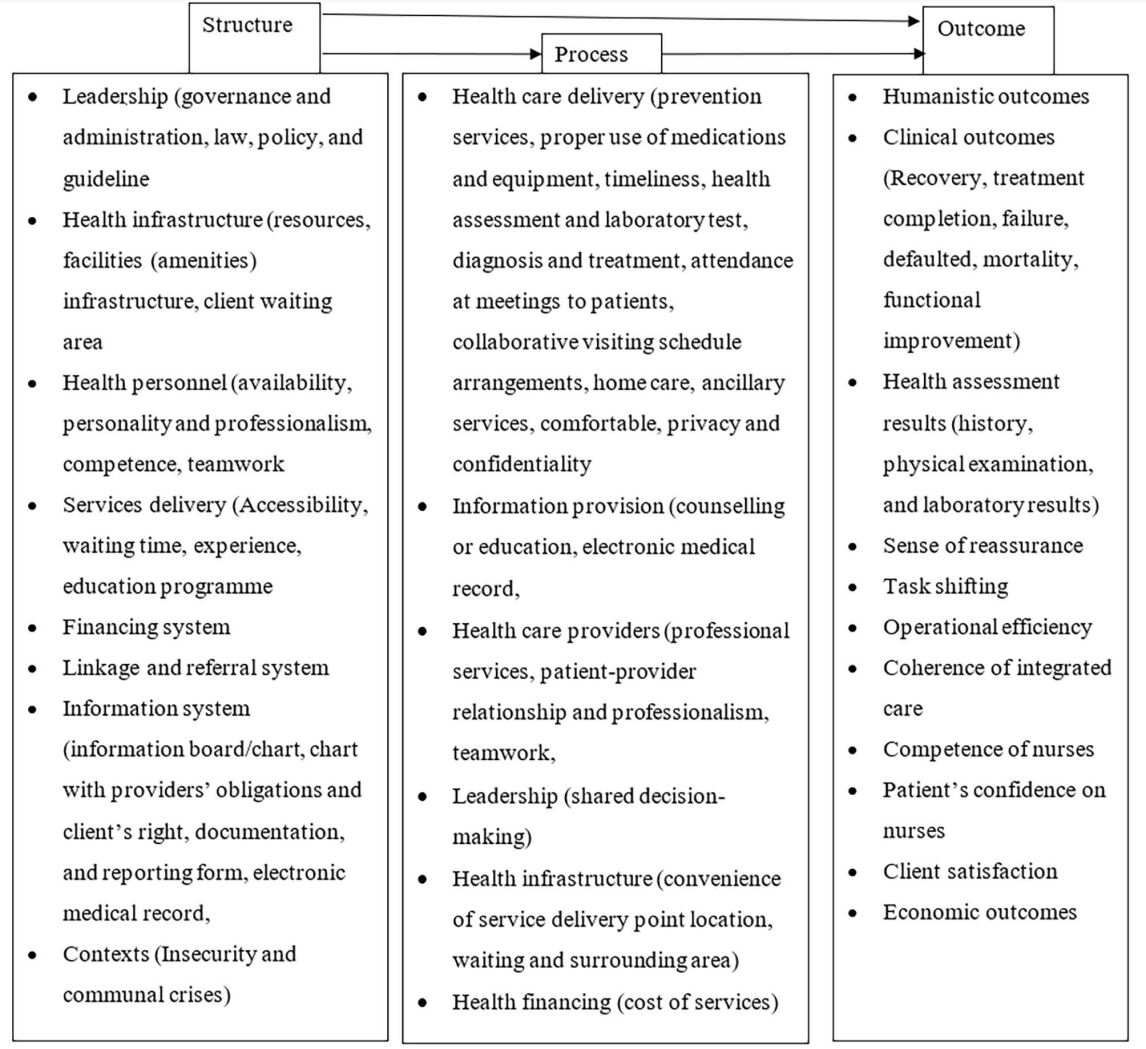
PHC quality indicators with their interaction based on Donabedian model

The common structure indicators were human resources for health, law and policy, infrastructure, facilities, and resources. Diagnosis (health assessment and/or laboratory tests) and management (health information, education, and treatment) were some of the process indicators. Clinical outcomes (cure, mortality, defaulter, treatment completion, recovery from pain) and satisfaction were the common measurement indicators of the outcome dimension. The main indicators based on the Donabedian quality care model are summarised in Fig. 2.

The details of each indicator with a citation are also shown in the supplementary file (supplementary file 2).

### Successes and challenges of quality of PHC

In addition to the identification of several indicators as determinants for the quality measure of PHC, the absence or presence of structure indicators, the appropriateness of process indicators, and the status of health service outcomes guide whether PHC is on a successful road map or struggling with challenges in the delivery of quality service. A similar level of perception between managers and clients on health care providers’ competency and professional conduct and a similar perception of clients and health care providers on structural factors (e.g., Nigeria) [45], high-quality structure indicators in some countries (e.g., Iran) [43], lower cause-specific mortality, and a lower rate of hospitalisation due to chronic disease and pneumonia in high-income countries (e.g., the USA) [37] were achievements. Challenges to quality PHC include high mortality due to tuberculosis in low-income countries (e.g., Uganda) [46], geographical disparity of quality care (e.g., Ethiopia and Iran) [40, 43], shortage of health care providers both in developed and developing countries, client and community engagement problems, lack of guidelines and providers’ poor adherence to guidelines [40], provision of inadequate information to clients [46], and poor quality due to a high admission rate (e.g., a mental disorder in the rural USA) [32] (Table 1). Table 1 shows the successes and challenges of quality of care in PHC based on the World Bank country categories.

**Table 1:** The successes and challenges of quality service delivery in PHC

Succusses	Challenges and/or unsuccessful progress
**Low-income countries (maternal and tuberculosis care)** • Folic acid supplementation, presence of measuring weight, accessibility and proper consultation time increased women’s satisfaction in Ethiopia [41] • Acceptable level (0.6%) of tuberculosis treatment failure in Uganda [46] **Lower-middle-income countries (general care and maternal service)** • Clients and health care provider’s similar perception to structural determinants in Nigeria [45]. • Satisfactory quality level of the structure dimension in the majority (86.4%) of the health centres; 95.4% of women were very satisfied with the services in Iran [43]. **Upper-middle-income country (general care)** • Patients and managers similar satisfaction level towards nurses’ competencies (87.6 vs. 85.7), professional conduct (86.2 vs. 85.7) and confidence in nurses (85.5 vs. 85.5) in South Africa [38]. **High-income countries (general care and pharmacy service)** • Lower population-level risk differences, lower cause specific mortality and lower rate of hospitalisations in the USA [37] • A positive impact of health support pharmacy service on outcome indicators, including clinical outcomes, humanistic outcomes, health behaviour change, community hub and impact on other professionals (sense of reassurance and operational efficiency) in Japan [34].	**Low-income countries (general care, maternal, adolescent and tuberculosis services)** • Disparity of quality of care between health centres in Ethiopia [40] • Medium level of quality (measured by satisfaction) for structure (58.8%), process (46.4%) and outcome (47.2%) indicators in adolescent and youth-friendly services in Ethiopia [40] • Unavailability of adequate and trained health care providers, poor care engagement of adolescents and youths, and lack of guidelines, protocols and procedures and providers’ poor adherence to guidelines in Ethiopia [40] • Only 55% of women were satisfied with ANC services in Ethiopia [41] • Inadequate information provision and health workers’ poor attitude to other health care providers (their fellow) in Uganda [46] • Lower percentage of treatment completion (40.3%), lower cure rate (39.2%), high mortality (6.8%) and a high percentage of defaulted treatment (12.5%) in tuberculosis case management in Uganda [46] **Lower-middle-income countries (general care and chronic disease services)** • Different satisfaction level of patients and managers to accessibility of care (96.3 vs. 85.7), supply of critical drugs (92.9 vs. 100), availability of equipment (97 vs. 57.2), friendliness (92.4 vs. 71.4) and attending to patients (74 vs. 57.2) in Nigeria [38] • Managers and patients complain about the poor quality of care due to long waiting times in Nigeria [39] • Insufficient manpower (40.3%), lack of basic amenities (light, water supply and good roads) (40.3%), insufficient equipment (18.1%), insecurity and communal crises (15.3%) and poor attitude of healthcare providers and clients in Nigeria [45] • Low mean scores for structure (34.5), process (38.5) and outcome (65.6) in Iran [42] • Lack of structure indicators and inappropriateness of process indicators in Iran [42] **Upper-middle-income country (general care and chronic disease services)** • Patients’ and managers’ different satisfaction level on health care provider’ coherence (97.4 vs. 85.7) in South Africa [38]. • Irregular pre-packing of drugs in South Africa [39] **High-income countries (mental and chronic disease services, and general care)** • Inappropriate use of restraints, catheters and psychoactive drugs in Canada [31] • High percentage of rural clinics lacking physicians and resources for preventive care of congestive heart failure, chronic obstructive pulmonary disease, diabetes, and bacterial pneumonia in the USA [37]. • Poor quality of rural mental health care in the USA [32] • Unrecognised impact of electronic health records on clinical outcome cross-developed countries [27]

## Discussion

This review summarised indicators, successes, and challenges of quality of care in PHC settings. Quality of PHC consists of an interaction of several quality indicators related to structure, process, and outcome, denoting physical and organisational characteristics where health care occurs and focusing on the care delivered to clients and the effect of health care on the status of patients and the population. The structure domain comprises health care resources, human resources, infrastructure, governance, law, policy, and guidelines. Providing preventive, professional, and ancillary services accompanied by professionalism was the common process indicator. Outcome indicators include mortality, cure rate, and treatment completion, behavioural change, and client satisfaction.

Quality of care indicators were identified. Some studies recruited quality indicators based on experts’ and health care providers’ perspectives [34–36] without community engagement. This may face feasibility, applicability, acceptability, implementation challenges, and a lack of comprehensiveness. For example, there was no structure indicator for geriatric pharmacotherapy [35]. This could be solved when perspectives from clients, families, health care providers, and administrators are considered. It is known that community engagement, continuous feedback, government support, and active community involvement play pivotal roles in the quality issues of PHC [49, 50], while a lesser client engagement decreased the quality of health care services [40]. Additionally, only one review assessed all quality elements (efficiency, effectiveness, safety, people-centredness, timeliness, equity, and integration) using structure, process, and outcome components [28] despite the importance of assessing the six domains of health care quality [25]. The Institute of Medicine has developed six domains of health care quality: safe, effective, patient-centred, timely, efficient, and equitable care [51]. The current review relies on previous studies, which did not present all domain of quality. Therefore, assessing the full domain of quality of PHC services under structure-process-outcome will give critical evidence.

The relationship between structure, process, and outcome indicators was a mediation process [38]. This was the direct and indirect relationship between structure, process, and outcome that worked when the outcome indicators were client satisfaction, coherence of integrated care, competence of nurses, and patients’ confidence in nurses. Clients were satisfied when they attended health institutions during convenient time, waited a short time to receive care, and attended a clean and suitable health institutions (e.g., waiting areas and other infrastructure). This means that clients were satisfied before interacting with health care providers, which indicates the need for critical attention during rating the status of the quality of care in the absence of process through which the real services are provided to clients. Studies investigated structure factors as the direct determinants of client satisfaction [52, 53]. Similarly, outcomes such as coherence of care and patient confidence in health care providers were affected by interpersonal aspects, shared decision-making procedures, and clients own problems and feelings [54].

Challenges persist in improving the quality of PHC services. Disparity of quality care between different health centres [40, 43] and a lack of structural inputs were reasons for the poor-quality care in low-income countries. There was also a low and varied quality of care between regions in middle-income countries due to the absence of support mechanisms, lack of coordination, problems in comprehensiveness and continuity of care [55–57], a lack of privacy and respect, an unsatisfactory pace of quality system development, and staff shortages [39, 58, 59]. Most countries have national quality care initiative strategies towards UHC [6], but they are not equally proactive in implementing the strategies. They also have different quality implementation approaches. For example, Donabedian’s system-based framework implementation is top-down in New Zealand and the UK, and bottom-up in Germany [23] though further research is indicated whether the top-down or bottom-up approach resulted in better quality of care. Countries may also have varied levels and extents of adapting PHC to different models of care, which the included articles did not address. Some are a ‘client circle of support’ [60], a ‘person-centred’ approach [61, 62], a ‘conversation approach’ [63], and ‘making or using action plans’ for PHC services [64].

Inadequate health workforces were understood challenge for poor quality care in low-income countries (e.g., Uganda) [46, 65]. For instance, the quality of ANC, adolescent, and youth-friendly service was low due to a shortage of adequate and trained health care providers. On the other hand, staff shortage was handled in such a way to do not interrupt the quality of care in high-income countries though workforce shortage was a challenge in developed countries. For example, the absence of physicians did not lower the quality of care in the USA [37]. The availability of other structure indicators and the substitution of the deficient personnel by other health care professionals could maintain high-quality care. For instance, a nurse-led PHC provided care equivalent to that of care by physician in chronic disease management [66], improved clinical outcomes and quality of life, and enhanced patient satisfaction [67, 68]. The health workforce shortage between developed and developing countries might vary based on the width and depth of health care. For example, the chiropractic workforce is unknown in some developing countries, and its shortage is sometimes underreported due to a poorly organised and unavailable written job description. In most developed countries, it is in practise, people demand the services, and the shortage can be reported [69]. Therefore, the health workforce shortage should be interpreted in light of the context.

Rate of admission was identified as a challenge for quality of PHC service delivery in rural area. For example, mental health care in rural settings was poor due to a lower chance of accessing appropriate care and an increasing admission rate in the USA [32]. This might be due to clients wait longer until they are seen by a health professional, and they might suffer from pain of disease progression if timely intervention is not provided.

Another challenge was a debate on electronic health records as one review reported that electronic health records have no impact on clinical outcomes [27]. However, another argument concluded that ‘electronic medical records improved quality of care, patient outcome and safety by improving management, preventing medical errors, reducing unnecessary investigations, and improving therapeutic interaction among primary care providers and patients [70]. Other studies also confirmed the importance of electronic medical records on quality of care improvement [71, 72] though there is a suggestion for a future prospective study [73].

This review has some limitations. Articles included in this review were conducted based on Donabedian’s quality framework. There may several articles have reported about quality of care. For example, there are factors that the current review did not address such as non-compassionate and unrespectful care can contribute to the low quality care because only 60% and 64% of health care providers provided compassionate and respectful care, for example, in Ethiopia despite caring, respectful, compassionate health care workers and quality included in the health care agenda [74, 75]. Similarly, in Uganda, a case study revealed that the national health system, overall working environment, national budgetary allocation to the health sector, and limited collaboration between health centres and hospitals are factors affecting the quality of health care [76]. Additionally, the articles included in this review were published only in English. There are articles published in non-English languages; including those articles may allow us to see the quality of PHC care in other countries contexts. Furthermore, the search was conducted only in four databases (Web of Science, Scopus, EMBASE, and PubMed) and Google Scholar. Other databases (e.g., Cochrane Library) may have related articles.

## Conclusions

Quality of care indicators varied according to the health care problems, which resulted in a disparity in the successes and challenges between developing and developed countries. Disparity in service coverage due to daily living conditions and mortality due to infectious diseases were more common in developing countries. On the other hand, quality of care problems due to chronic diseases were recorded in developed countries. Inadequate health workforce was a challenge in developing and developed countries as a structure component of quality care provision. The PHC system should ensure the presence of adequate health care providers, equipped health care facilities, compliance with health policy and laws, adequate financing, and enhanced community and client participation. Additionally, each country should implement national quality initiative strategies with appropriate monitoring and evaluation of performance in each structure, process, and outcome indicator. PHC quality improvement needs appropriate resources and infrastructure, and an adequate PHC workforce with skill mix.

### Electronic supplementary material

Below is the link to the electronic supplementary material.

Supplementary Material 1

Supplementary Material 2

## Data Availability

The data set is available within this manuscript.

## Abbreviations

CHWs: Community Health Workers
PHC: Primary Health Care
UK: United Kingdom
UHC: Universal Health Coverage
UNs: United Nations
USA: United States of America
WHO: World Health Organisation
