# Supplementary material for: Successes and challenges towards improving quality of primary health care services: a scoping review
Source: BMC Health Serv Res. 2023 Aug 23;23:893. doi: 10.1186/s12913-023-09917-3 (PMC10464348; doi:10.1186/s12913-023-09917-3)
Supplement: Supplementary file 1 — Supplementary Material 1 [file 12913_2023_9917_MOESM1_ESM.docx]

Supplementary file 1: Search strategy

| Databases | | Search string | Number of articles |
| --- | --- | --- | --- |
| PubMed | (((((“primary health care”[All Fields] OR “primary healthcare”[All Fields] OR “primary care”[All Fields]) AND “quality of care”[All Fields]) OR “quality”[All Fields] OR “quality care”[All Fields] OR “quality of health care”[All Fields] OR “quality of healthcare”[All Fields]) AND “Donabedian”[All Fields]) OR “Donabedian’s model”[All Fields] OR “Donabedian’s structure process outcome”[All Fields] OR “Donabedian model”[All Fields]) OR “Donabedian structure process outcome”[All Fields] OR “Donabedian’s structure-process-outcome”[All Fields] OR “Donabedian structure-process-outcome”[All Fields] OR “structure-process-outcome”[All Fields] | | 722 |
| Web of Science | | | |
| #1 (Step 1) | ((ALL=("primary health care")) OR ALL=("primary healthcare")) OR ALL=("primary care") | | 281,069 |
| #2 (Step 2) | ((((ALL=("quality of care")) OR ALL=(quality)) OR ALL=("quality care")) OR ALL=("quality of health care")) OR ALL=("quality of healthcare") | | 3,477,432 |
| #3 (Step 3) | (((((((ALL=(donabedian)) OR ALL=("donabedian's model”)) OR ALL=("donabedian's structure process outcome")) OR ALL=("donabedian model")) OR ALL=("donabedian structure process outcome")) OR ALL=("donabedian's structure-process-outcome")) OR ALL=("donabedian structure-process-outcome")) OR ALL=("structure-process-outcome") | | 1,091 |
| Final search strategy ((#1 AND #2) AND #3 | ((ALL=("primary health care")) OR ALL=("primary healthcare")) OR ALL=("primary care") AND ((((ALL=("quality of care")) OR ALL=(quality)) OR ALL=("quality care")) OR ALL=("quality of health care")) OR ALL=("quality of healthcare") AND (((((((ALL=(donabedian)) OR ALL=("donabedian's model”)) OR ALL=("donabedian's structure process outcome")) OR ALL=("donabedian model")) OR ALL=("donabedian structure process outcome")) OR ALL=("donabedian's structure-process-outcome")) OR ALL=("donabedian structure-process-outcome")) OR ALL=("structure-process-outcome") | | 78 |
| EMBASE | | | |
| #1 | 'primary health care'/exp OR 'primary health care' OR 'primary healthcare' OR 'primary care' | | 367,713 |
| #2 | 'quality of care'/exp OR 'quality of care' OR quality OR 'quality care' OR 'quality of health care' OR 'quality of healthcare' | | 2,368,679 |
| #3 | donabedian OR ‘donabedian model’ OR ‘donabedian structure process outcome’ OR ‘donabedian structure-process-outcome’ OR ‘structure process outcome’ | | 1,155 |
| Final search strategy ((#1 AND #2) AND #3 | ('primary health care'/exp OR 'primary health care' OR 'primary healthcare' OR 'primary care') AND ('quality of care'/exp OR 'quality of care' OR quality OR 'quality care' OR 'quality of health care' OR 'quality of healthcare') AND (donabedian OR ‘donabedian model’ OR ‘donabedian structure process outcome’ OR ‘donabedian structure-process-outcome’ OR ‘structure process outcome’) | | 84 |
| SCOPUS | | | |
| #1 | ( TITLE-ABS-KEY ( "primary health care" ) OR TITLE-ABS-KEY ( "primary healthcare" ) OR TITLE-ABS-KEY ( "primary care" ) ) | | 226,757 |
| #2 | ( TITLE-ABS-KEY ( "quality of care" )  OR  TITLE-ABS-KEY ( quality )  OR  TITLE-ABS-KEY ( "quality care" )  OR  TITLE-ABS-KEY ( "quality of health care" )  OR  TITLE-ABS-KEY ( "quality of healthcare" ) ) | | 4,461,195 |
| #3 | ( TITLE-ABS-KEY ( donabedian )  OR  TITLE-ABS-KEY ( "donabedian's model" )  OR  TITLE-ABS-KEY ( "donabedian model" )  OR  TITLE-ABS-KEY ( "donabedian's structure process outcome" )  OR  TITLE-ABS-KEY ( "donabedian structure process outcome" )  OR  TITLE-ABS-KEY ( "donabedian's structure-process-outcome" )  OR  TITLE-ABS-KEY ( "donabedian structure-process-outcome" )  OR  TITLE-ABS-KEY ( "structure process outcome" ) ) | | 839 |
| Final search strategy #1 AND #2 AND #3 | ( TITLE-ABS-KEY ( "primary health care" ) OR TITLE-ABS-KEY ( "primary healthcare" ) OR TITLE-ABS-KEY ( "primary care" ) ) AND ( TITLE-ABS-KEY ( "quality of care" )  OR  TITLE-ABS-KEY ( quality )  OR  TITLE-ABS-KEY ( "quality care" )  OR  TITLE-ABS-KEY ( "quality of health care" )  OR  TITLE-ABS-KEY ( "quality of healthcare" ) ) AND ( TITLE-ABS-KEY ( donabedian )  OR  TITLE-ABS-KEY ( "donabedian's model" )  OR  TITLE-ABS-KEY ( "donabedian model" )  OR  TITLE-ABS-KEY ( "donabedian's structure process outcome" )  OR  TITLE-ABS-KEY ( "donabedian structure process outcome" )  OR  TITLE-ABS-KEY ( "donabedian's structure-process-outcome" )  OR  TITLE-ABS-KEY ( "donabedian structure-process-outcome" )  OR  TITLE-ABS-KEY ( "structure process outcome" ) ) | | 66 |
