# Supplementary material for: Successes and challenges towards improving quality of primary health care services: a scoping review
Source: BMC Health Serv Res. 2023 Aug 23;23:893. doi: 10.1186/s12913-023-09917-3 (PMC10464348; doi:10.1186/s12913-023-09917-3)
Supplement: Supplementary file 2 — Supplementary Material 2 [file 12913_2023_9917_MOESM2_ESM.docx]

Supplementary file 2: Indicators in the Donabedian’s structure-process-outcome measure of the quality of PHC

| Structure factors | Process factors | Outcome |
| --- | --- | --- |
| Law, policy and guideline, and administration (1-6) | Prevention services (7) | Humanistic outcomes (3, 4) |
| Facilities (amenities) and infrastructure (1, 5, 6, 8, 9) | Pre-packing drugs (10) | Clinical outcomes (3, 4, 6, 11) |
| Governance and accountability (11) | Information, counselling or education (1, 3, 4, 8, 11-13) | Economic outcomes(3, 4) |
| Resources (2, 3, 6, 11) | Waiting date to receive laboratory test result (13) | Sense of reassurance (3, 4) |
| Technical provision (11) | Waiting date to receive drugs (13) | Task shifting (3) |
| Practice integration (11) | Attendance at meetings to patients (3, 14) | Operational efficiency (3, 4) |
| Accessibility (11, 12, 14) | Collaborative visiting schedule arrangements (3, 4) | Health assessment results (history, physical examination and laboratory results) (8) |
| Organisational structure (11) | Withdrawal of home pharmaceutical care (3) | Cure rate (13) |
| Waiting time (12) | Home pharmaceutical care pathway (3) | Treatment completion (6, 13, 15) |
| Human resource (2, 3, 5, 6, 8-10, 13, 16) | Non-professional or ancillary services (1, 3) | Treatment failure (13) |
| Competence (3, 4) | Timeliness (3) | Defaulted treatment (13) |
| Remuneration system or budget (3, 5) | Grief care (3) | Mortality (13) |
| Equipment and drugs (5, 8-10, 14) | Professional services (3, 4) | Recovered from pain (12) |
| Insecurity and communal crises (9) | Use of equipment and drugs (16) | Functional improvement (12) |
| Documenting and reporting form (8) | Patient-provider relationship/interaction (1, 11) | Health behaviour change (4) |
| Referral system (5, 8) | Immunisation use (8) | Community hub (4) |
| Electronic medical record (17) | Taking treatment (11) | Tooth brushing coverage (15) |
| Teamwork (3, 4) | Follow-up (11) | Treatment remission at 6 or 12 months (11) |
| Recognition of benefits of home Pharmaceutical care (3) | Health assessment and laboratory test (8, 11, 12) | Competence (14) |
| Experience (3, 4) | Shared decision-making (11) | Confidence (14) |
| Education program (3) | Therapeutic exercise (12) | Dental urgency (15) |
| Pharmacists’ authority (3) | Teamwork (4) | Recall (6) |
| A sign board that mentions operating hours and date (5) | Electronic medical record (17) | Client satisfaction (1, 5, 6, 18, 19) |
| Separate space to services and waiting area (5) | Convenience of service opening hours (5) |  |
| Availability of information document in waiting area (5) | Waiting time (5) | Long waiting time (10) |
| Updated lists of services, providers’ obligations and clients right included in the package (5) | Friendliness of supportive staffs and health workers (5, 14) |  |
| Professionalism (3, 4) | Attitude of clients and health workers (9) | Coherence (14) |
| Personality (3, 4) | Waiting area comfortableness (5) |  |
|  | Privacy and confidentiality protection during the consultation (5) |  |
|  | Length of time of consultation (5) |  |
|  | Freedom of asking health care providers (5) |  |
|  | Understanding of information given by health care provider (5) |  |
|  | Cost of services (5) |  |
|  | Diagnosis, treatment procedure/plan (5, 6) |  |
|  | Adequacy of psychosocial assessment (5) |  |
|  | Convenience of service delivery point location (5) |  |
|  | Professionalism, like respectfulness (5, 14) |  |
|  | Cleanliness of areas surrounding health facility (5) |  |
|  | Management (6) |  |
|  | Assistive device (12) |  |
|  | Surgery (12) |  |
|  | Gastroprotection (12) |  |
|  | First-line pharmacological treatment (12) |  |
|  | Assessment for use of anti-inflammatory medications (12) |  |
|  | Records (6) |  |
|  | Weight loss (12) |  |

**Reference for supplementary file 2**

1. Zhou J, Blaylock R, Harris M. Systematic review of early abortion services in low- and middle-income country primary care: potential for reverse innovation and application in the UK context. Global Health. 2020;16(1):91.

2. Sato N, Fujita K, Kushida K, Chen TF. Exploring the factors influencing the quality of “Health Support Pharmacy” services in Japan: Perspectives of community pharmacists. Research in Social and Administrative Pharmacy. 2020;16(12):1686-93.

3. Fujita K, Kushida K, Moles RJ, Chen TF. Home healthcare professionals’ perspectives on quality dimensions for home pharmaceutical care in Japan. Geriatrics & Gerontology International. 2019;19(1):35-43.

4. Sato N, Fujita K, Kushida K, Chen TF. Exploring the factors influencing the quality of "Health Support Pharmacy" services in Japan: Perspectives of community pharmacists. Res Social Adm Pharm. 2020;16(12):1686-93.

5. Gebrie M, Asrade G, Tsehay CT, Yazachew L, Dellie E. Quality of adolescent and youth-friendly health services in Dehana district public health facilities, northeast Ethiopia: Using the Donabedian quality framework. PLoS ONE. 2021;16(10 October 2021).

6. Gardner K, Mazza D. Quality in general practice: Definitions and frameworks. Australian Family Physician. 2012;41(3):151-4.

7. Agiro A, Wan TT, Ortiz J. Organizational and Environmental Correlates to Preventive Quality of Care in US Rural Health Clinics. J Prim Care Community Health. 2012;3(4):264-71.

8. Noori Z, Khorasani P, Hosseini H. Application of Donabedian framework of structure, process and outcome in diabetes management among elderlies living in nursing homes in Isfahan, Iran.

9. Uzomba AE, Ndep AO, Uzomba CI, Ekpenyong BN, Edom AE. Factors influencing the quality of primary healthcare services in southern senatorial district of cross river state, nigeria. Indian Journal of Public Health Research and Development. 2021;12(2):426-31.

10. Ameh S, Klipstein-Grobusch K, D'Ambruoso L, Kahn K, Tollman SM, Gómez-Olivé FX. Quality of integrated chronic disease care in rural South Africa: user and provider perspectives. Health Policy Plan. 2017;32(2):257-66.

11. Petrosyan Y, Sahakyan Y, Barnsley JM, Kuluski K, Liu B, Wodchis WP. Quality indicators for care of depression in primary care settings: a systematic review. Syst Rev. 2017;6(1):126.

12. Petrosyan Y, Sahakyan Y, Barnsley JM, Kuluski K, Liu B, Wodchis WP. Quality indicators for care of osteoarthritis in primary care settings: a systematic literature review. Fam Pract. 2018;35(2):151-9.

13. Bulage L, Sekandi J, Kigenyi O, Mupere E. The quality of tuberculosis services in health care centres in a rural district in Uganda: the providers' and clients' perspective. Tuberc Res Treat. 2014;2014:685982.

14. Ameh S, Gómez-Olivé FX, Kahn K, Tollman SM, Klipstein-Grobusch K. Relationships between structure, process and outcome to assess quality of integrated chronic disease management in a rural South African setting: applying a structural equation model. BMC Health Serv Res. 2017;17(1):229.

15. Viana IB, Moreira RD, Martelli PJD, de Oliveira ALS, Monteiro ID. Evaluation of the quality of oral health care in Primary Health Care in Pernambuco, Brazil, 2014. Epidemiologia E Servicos De Saude. 2019;28(2).

16. Hillmer MP, Wodchis WP, Gill SS, Anderson GM, Rochon PA. Nursing home profit status and quality of care: is there any evidence of an association? Med Care Res Rev. 2005;62(2):139-66.

17. Holroyd-Leduc JM, Lorenzetti D, Straus SE, Sykes L, Quan H. The impact of the electronic medical record on structure, process, and outcomes within primary care: a systematic review of the evidence. Journal of the American Medical Informatics Association. 2011;18(6):732-7.

18. Hussen MA, Worku BT. Quality of Antenatal Care Service and Factors Associated with Client Satisfaction at Public Health Facilities of Bele Gasgar District. J Patient Exp. 2022;9:23743735221083163.

19. Sardasht FG, Shourab NJ, Jafarnejad F, Esmaily H. Comparing the quality of preconception care provided in healthcare centers in Mashhad in 2012. Electron Physician. 2015;7(2):1039-46.
